# Wireless Body Area Network Control Policies for Energy-Efficient Health Monitoring

**DOI:** 10.3390/s21124245

**Published:** 2021-06-21

**Authors:** Yair Bar David, Tal Geller, Ilai Bistritz, Irad Ben-Gal, Nicholas Bambos, Evgeni Khmelnitsky

**Affiliations:** 1 Department of Industrial Engineering, Tel Aviv University, Tel-Aviv 69978, Israel; yairbd2@gmail.com (Y.B.D.); taljgeller@gmail.com (T.G.); bengal@tauex.tau.ac.il (I.B.-G.); xmel@tau.ac.il (E.K.); 2 Department of Electrical Engineering, Stanford University, Stanford, CA 94305, USA

**Keywords:** wireless body area networks, controlled sensing, energy efficiency, partially observable Markov decision processes (POMDPs), remote health monitoring

## Abstract

Wireless body area networks (WBANs) have strong potential in the field of health monitoring. However, the energy consumption required for accurate monitoring determines the time between battery charges of the wearable sensors, which is a key performance factor (and can be critical in the case of implantable devices). In this paper, we study the inherent trade-off between the power consumption of the sensors and the probability of misclassifying a patient’s health state. We formulate this trade-off as a dynamic problem, in which at each step, we can choose to activate a subset of sensors that provide noisy measurements of the patient’s health state. We assume that the (unknown) health state follows a Markov chain, so our problem is formulated as a partially observable Markov decision problem (POMDP). We show that all the past measurements can be summarized as a belief state on the true health state of the patient, which allows tackling the POMDP problem as an MDP on the belief state. Then, we empirically study the performance of a greedy one-step look-ahead policy compared to the optimal policy obtained by solving the dynamic program. For that purpose, we use an open-source Continuous Glucose Monitoring (CGM) dataset of 232 patients over six months and extract the transition matrix and sensor accuracies from the data. We find that the greedy policy saves ≈50% of the energy costs while reducing the misclassification costs by less than 2% compared to the most accurate policy possible that always activates all sensors. Our sensitivity analysis reveals that the greedy policy remains nearly optimal across different cost parameters and a varying number of sensors. The results also have practical importance, because while the optimal policy is too complicated, a greedy one-step look-ahead policy can be easily implemented in WBAN systems.

## 1. Introduction

WBANs enable the estimation of the physical and health condition of a patient with unprecedented precision as well as the ability to monitor the evolution of these heath conditions over time [1,2,3,4,5]. The control of WBANs involves activating a set of sensors that are located on or near a patient (e.g., wearable) while collecting data related to their physiological and mental activities. The sensing process is controlled by a computing unit, which selects a subset of sensors to be activated at a given time, analyzes the obtained measurements, and transmits the information and initial results to a central unit (server) for further analysis and decision making.

Recent research and practical developments in sensors and WBAN technology allow efficient implementations of WBANs in modern real-world applications [3,4,6]. One of the main factors that impacts the performance of such systems in the context of health monitoring is the inherent trade-off between minimizing the energy consumption of operating the sensors and maximizing the accuracy of the health state estimation based on the measured information [1,2]. In this paper, we focus on this trade-off between energy costs and accuracy and propose a tractable model that allows designing efficient algorithms that balance this trade-off. 

Our goal is to design an efficient dynamic policy that the controller will use to select a subset of sensors at each epoch, which maintains highly accurate knowledge of the patient’s health state while saving as much energy as possible. 

We formulate a dynamic health-sensing model based on partially observable Markov decision processes (POMDPs). A POMDP is defined by a set of states, a set of actions, the transition probabilities between states, a cost function, and a set of observations [7]. 

To convert our POMDP into an MDP, we show that all the information captured in all the past outputs can be summarized using a belief vector, which is a probability vector over the possible health states. This belief state is the state of our MDP. The analysis of the MDP essentially allows computing the optimal policy for the problem by means of dynamic programming. 

By discretizing the continuous belief state, we can numerically approximate the optimal policy for our dynamic program with an arbitrary degree of accuracy. However, computing the optimal policy has a very high computational complexity that renders it impractical. Even if the optimal policy can be computed offline given the patient parameters, it requires storing very large policy tables on the controller, which should be a simple and efficient device. An alternative is the greedy one-step look-ahead policy, which is generally a suboptimal compromise. Its performance compared to the optimal policy depends on the problem parameters, among them the transition matrix between the health states, the sensor accuracies, and the cost function parameters. 

We study the empirical performance of the greedy policy using an open-source Continuous Glucose Monitoring (CGM) dataset [8], which includes the measurements of 232 patients from different age groups and backgrounds over ≈6 months. We estimate the transition matrix and sensor accuracies from the data and then evaluate the greedy policy over these 232 patients. Our empirical study includes sensitivity analysis for the cost parameters and the number of sensors that are external to the data. We also study the performance of the greedy policy using synthetic simulations over a wider range of parameters.

Our results show that while the greedy algorithm is suboptimal in general, it is an appealing choice for CGM. The greedy policy saves ≈50% of the energy costs while reducing the accuracy cost by less than 2% (i.e., reducing the numerical cost by a factor of 0.98 and not the accuracy itself). Our results are encouraging, since they suggest that in certain cases, the simple and efficient greedy policy can be used for WBAN control in practice while losing very little compared to the optimal intractable policy. 

### 1.1. Related Work

Optimization techniques for WBANs regularly deal with optimizing the hardware and communication components, such as wireless communication protocols between the sensors and the controlling unit, to reduce energy consumption [9,10,11]. Another stream of research has aimed to increase energy efficiency by optimizing the controlling algorithms, which usually concern sensor selection based on the information gained through the system’s activities [3,12], or optimizing the resource allocation in the system [13,14,15]. Another important aspect of designing a WBAN system is to ensure the confidentiality and integrity of the data, since any malicious attempt on the data the WBAN system collects can have dire consequences [5,16].

To improve the energy efficiency of the WBAN system, it is also important to reduce the volume of the transmitted data, which has been the focus of several studies [16,17,18,19]. In contrast, we focus on the energy costs of sensing and operating the sensors. To design a full system, our approach can be combined with that of [2,4,20,21] in a modular manner.

POMDPs have been used to model the behavior and information transitions in WBANs [2,4,20,21]. Prior work on WBAN control used simplifying assumptions such as allowing only homogeneous or identical sensors and “perfect sensing information” [7,9,11]. We extend the POMDP model to allow multiple non-identical noisy sensors, which yield a vector of sensor outputs at each epoch. 

### 1.2. Outline

The rest of the paper is organized as follows. Section 2 formulates our health sensing Markov model and our joint energy-accuracy objective. Section 3 uses the belief state approach to convert our POMDP problem to a MDP problem. Section 4 utilizes the convenient MDP form to propose a one-step look-ahead policy, and it also shows how to compute the optimal policy, which serves as a benchmark to the proposed policy. Section 5 provides the empirical analysis of the proposed approach tested on a real-time Continuous Glucose Monitoring (CGM) dataset. Section 6 provides extended empirical results, which include sensitivity analysis several generalizations of our model.

## 2. Health Sensing Model

### 2.1. Patient Health States

We first discuss the health state transitions. We assume a finite set of patient health states, denoted by$\;ℋ=\{h_{1},h_{2},\ldots ,\;h_{J}\}$. The health states can represent the process of a given disease or different health conditions or a mixture of both. If the health states represent severity levels of a disease, then the set ℋ will be ordered such that for each pair $h_{j},h_{j^{\prime }}$ where $j<j^{\prime }$, $h_{j}$ is considered less healthy than $h_{j^{\prime }}$. Accordingly, $h_{J}$ is considered the healthiest state, and $h_{1}$ is considered the least healthy state. In some applications, $h_{1}$ can be considered a terminal health state in which the monitoring of the individual is no longer relevant (e.g., hospitalization, patient mortality, etc.). If the health states represent different conditions, then there is no simple order between them; for example, if the states are swine flu, COVID-19, and an allergic response.

We assume that the health state follows a discrete-time Markov chain, and we call each time step t an epoch, which is the time steps when the controller makes a decision. The transition probability between any two states during two consecutive epochs, $h^{t},h^{t+1}\in ℋ,$ is given by a transition matrix T, in which the (i,j) element is given by:

(1) $$T_{ij}=Pr(h^{t+1}=h_{j}|h^{t}=h_{i}).$$

In practice, the transition probabilities can be estimated using historical clinical data. Therefore, we assume that T is known to the controller and is fixed throughout the sensing (monitoring) period. 

### 2.2. Sensors

Consider a WBAN that consists of N sensors operating over a time horizon of L epochs. Each sensor may be responsible for collecting different types of information (e.g., different vital signs). The information collected by the sensors is used to estimate the patient’s health state, which is unknown to the controller at all times. At each decision epoch, t, the controller chooses to activate some subset of the N sensors from the action set S of all possible activation subsets. Since some combinations of sensors cannot be realized due to physical or physiological limitations, the size of S might be smaller than $2^{N}$. Naturally, reducing the size of the set S reduces the complexity of the controller’s decision making. The sensor activation status at epoch t is denoted by $s^{t}=\left(s_{1}^{t},\;s_{2}^{t},\ldots ,s_{N}^{t}\right)$, where $s_{n}^{t}=1$ refers to an activated sensor, and $s_{n}^{t}=0$ refers to a deactivated sensor, for n=1,…,N. The active sensors are selected to optimize a cost function (defined in Section 2.3) that considers both the energy consumption and misclassification costs. For ease of notation, the time index may be omitted when discussing the general time-independent properties.

Let $l_{n}^{t}$ denote the output of sensor *n* at time t, while $l^{t}$ is the output vector of all the sensors at t, $L\left(s^{t}\right)$ is the set of all the possible output vectors for a sensor activation vector$\;s^{t}$, and ${\left\{l^{t}\right\}}_{t=1}^{E}$ is a sequence of sensor outputs during E epochs. We assume that given a patient’s health state, the probability of a certain sensor output is known. For example, given the particular health state of a patient with diabetes, the probability that a blood sugar level sensor will return a certain value may be obtained from a known distribution.

WBAN sensors are typically power-constrained and low-cost. To account for this, we consider detectors that can only sense specific threshold crossings. Hence, each sensor can only provide output from a set of discrete outputs with size Λ. While any number of thresholds is possible in our model, we are mainly motivated by a small number of thresholds (Λ=2,3), since then, the sensors are much simpler to implement and operate, which reduces their battery consumption. 

For example, if Λ=3, then $l_{n}^{t}\in \left\{0,1,2,\emptyset \right\}$, where ∅ denotes a deactivated sensor. Suppose that there are three sensors,$\;s_{1},s_{2}\;\mathrm{and}\;s_{3}$, and only $s_{1}$ and $s_{2}$ have been activated, so s=(1,1,0). Then, L(s)={(2,2,∅),(2,1,∅),(2,0,∅),(1,2,∅),(1,1,∅),(1,0,∅),(0,2,∅),(0,1,∅),(0,0,∅)}.

We define $A_{n}^{\Lambda \times J}$ as the output probabilities matrix. Each component $A_{n}{}_{ij}$ expresses the probability of observing output “i” from sensor n given that the individual is currently in health state $h_{j}$:

(2) $$A_{n}{}_{ij}=Pr\left(l_{n}=i|h_{j}\right)\;n=1,\ldots ,N;i=0,\ldots ,\Lambda -1\;;j=1,\ldots ,J.$$

**Definition** **1.** *Define the output function of sensor n as:*

(3) $$f_{n}\left(l_{n},j\right)=Pr\left(l_{n}\;|\;h=h_{j}\right)=\{\begin{array}{cc} A_{n}{}_{\left(\Lambda -1\right)j} & l_{n}=\Lambda -1\\ \ldots & \ldots \\ A_{n}{}_{1j} & l_{n}=1\\ A_{n}{}_{0j} & l_{n}=0\\ 1 & l_{n}=\emptyset \end{array}.$$

Note that since a deactivated sensor ($l_{n}=\emptyset$) provides no information, its output function should return 1 in order to neutralize its impact on the probability of the output of the activated sensors.

We assume independence between the sensor outputs given a patient’s true health state. This is a natural assumption, since it is only the health state that couples the sensors’ outputs, and each is affected by its own local noise. Therefore, we may define the output function of all sensors as:

(4) $$f\left(l,j\right)=Pr\left(l\;|\;h=h_{j}\right)=\prod_{n=1}^{N}Pr\left(l_{n}\;|\;h=h_{j}\right)=\prod_{n=1}^{N}f_{n}\left(l_{n},j\right).$$

### 2.3. Power and Misclassification Costs

Our goal is to develop algorithms that account for both the cost of activating a set of sensors and the misclassification cost associated with possible errors in determining the patient’s health state. The cost of activating a set of sensors, s, is denoted by C(s). The formulation of the misclassification cost is often more complicated and is related to the false positive and false negative classification errors. If the patient’s actual health state is better than the estimated one, the system incurs a false positive error cost, which is proportional to the probability of such error. Similarly, the false negative error cost is proportional to the probability that the actual heath state of the patient is worse than the estimated one. We denote the false positive and false negative cost parameters by $C_{FP}$ and $C_{FN}$, respectively. Since the actual health state is unknown and random, the system may incur both false positive and false negative costs at the same time. The following definition formulates the considered misclassification cost.

**Definition** **2.** *The misclassification cost per health state,*$h_{j}$*, denoted by* $\rho_{j}\left({\left\{l^{\tau }\right\}}_{\tau =1}^{t}\right)$*, is defined as:*

(5) $$\rho_{j}\left({\left\{l^{\tau }\right\}}_{\tau =1}^{t}\right)=C_{FP}\sum_{i=1}^{j-1}Pr\left(h^{t+1}=h_{i}\;|\;{\left\{l^{\tau }\right\}}_{\tau =1}^{t}\right)+C_{FN}\sum_{i=j+1}^{J}Pr\left(h^{t+1}=h_{i}\;|{\left\{l^{\tau }\right\}}_{\tau =1}^{t}\right).$$

As indicated above, we multiply the probability that the patient is in a state worse than a specific health state $h_{j}^{t}$ by the false-positive cost parameter $C_{FP}$, and we multiply the probability that the patient is in a state better than $h_{j}^{t}$ by the false negative cost parameter $C_{FN}$. A more sophisticated cost structure is possible. In particular, a health state’s specific costs and/or dynamic costs can be incorporated in the proposed model. 

Then, the total estimated misclassification cost is defined as:

(6) $$\rho \left({\left\{l^{\tau }\right\}}_{\tau =1}^{t}\right)=\sum_{j=1}^{J}Pr\left(h^{t+1}=h_{j}\;|\;{\left\{l^{\tau }\right\}}_{\tau =1}^{t}\right)\cdot \rho_{j}\left({\left\{l^{\tau }\right\}}_{\tau =1}^{t}\right).$$

### 2.4. Motivating Example 

**Figure 1:**
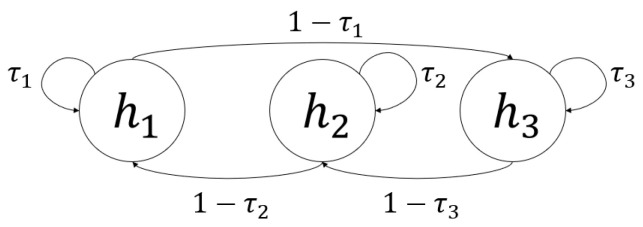
Schematic description of the Markov chain.

As we show in Section 6, by wisely choosing the subset of sensors to activate, much energy can be saved with only a small reduction in the average misclassification cost compared to activating all sensors. In this subsection, we illustrate this idea given a simple example. We examine a scenario where patient’s health condition can be classified into one of three health states, $ℋ=\left\{h_{1},\;h_{2},\;h_{3}\right\}$. We assume that for each epoch t, the patient may either transit to the neighboring less health state or remain in the current state. For $h_{1}$, the patient may either remain in $h_{1}$ or transit to the healthiest state $h_{3}$. The Markov chain is depicted in Figure 1, for some probabilities $0<\tau_{1},\;\tau_{2},\tau_{3}<1$. 

We also assume three completely accurate sensors with possible outputs {0,1,∅}, where each sensor can perfectly detect a particular health state. The sensors outputs probabilities matrices are:

$$A_{1}=\left[\begin{array}{ccc} 0 & 1 & 1\\ 1 & 0 & 0 \end{array}\right]\;\;\;\;\;A_{2}=\left[\begin{array}{ccc} 1 & 0 & 1\\ 0 & 1 & 0 \end{array}\right]\;\;\;\;\;A_{3}=\left[\begin{array}{ccc} 1 & 1 & 0\\ 0 & 0 & 1 \end{array}\right].$$

If the patient is in state $h_{1}$, the first sensor would output a signal “1”, while the other two sensors would output “0”. Similarly, if the patient is in state $h_{2}$ or $h_{3}$, the corresponding sensor would output “1”, while the other two would output “0”.

The optimal policy in this scenario only requires activating a single sensor throughout the sensing period (except for at $t_{0}$) and still provides perfectly accurate sensing. At first, the controller activates all sensors to identify the initial health state of the patient. From there on, at each epoch, the controller only activates the sensor that corresponds to the current health state to identify whether the patient has transitioned to the next health state or remained in the current health state. Compared to the naïve solution of constantly activating all sensors, the optimal solution results in a significant reduction of energy consumption (a single sensor is activated) while preserving perfect knowledge of the patient’s health state. Even with imperfect but still fairly accurate sensors, activating a single sensor in this manner will result in a small misclassification cost while reducing the energy consumption dramatically. 

The optimal policy in the above scenario is intuitive and easy to guess. Once the sensor output probabilities matrix is not deterministic or the transition matrix has a different structure, computing the optimal solution amounts to solving the POMDP, which in general is highly complicated. As done in the literature, the first step to solve the POMDP is to summarize the information captured in the past measurements using a compact belief state on the health state of the patient. This converts the POMDP into a belief state MDP for which we can, in principle, compute the optimal policy by dynamic programming. 

## 3. Belief States 

The patient’s actual health state is unknown to the controller, and our goal is to monitor and estimate this state. Therefore, the controller uses the sensors’ outputs to produce a probability distribution over the set of health states. This distribution is defined as a belief state.

**Definition** **3.** *The belief state* $b^{t}=\left(b_{1}^{t},\ldots ,b_{J}^{t}\right)$*is definedsuch that* $\;b_{j}^{t}=Pr\left(h^{t}=h_{j}\;|\;{\left\{l^{\tau }\right\}}_{\tau =1}^{t}\right)$ *for every j.*

**Figure 2:**
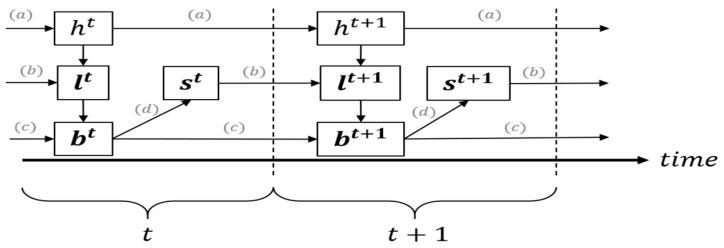
Order of events throughout sensing epochs.

The belief state is the probability distribution of the health states of the patient given the past measurements. Next, we prove that tracking belief states instead of all past measurements comes with no loss of optimality (i.e., it is a sufficient statistic) and that it can be recursively updated. During the monitoring period, the belief states evolve based on the outputs, $l^{t},\;$received from the activated sensors, $s^{t-1}$. Below is a schematic description of the order of events in two specific subsequent epochs:

As shown in Figure 2, (a) health states $h^{t}$ evolve according to the Markov chain transition matrix as an independent process. (b) The subset of activated sensors $s^{t}$ at each epoch provides outputs $l^{t+1}$ during the following epoch t+1. (c) The activated sensor outputs also depend on the health state. Using the activated sensor outputs $l^{t+1}$ and the belief state $b^{t}$, we update the belief state $b^{t+1}$. (d) Finally, given the updated belief state, the controller selects the subset of activated sensors $s^{t+1}\;$at the next epoch.

### Markovian Property of Belief States

In this subsection, we show that the past sensor measurements can be summarized as a belief state vector, which is a Markov chain. This converts the POMDP into a belief state MDP. Note that in contrast to a generic POMDP, our cost function is by itself an average taken over the belief states.

**Lemma** **1.** *The belief state* $b^{t}$*is Markovian. For any* 0≤b≤1*, the transition probabilities are given by*

(7) $$Pr\left(b_{j}^{t+1}\;=b|\;{\left\{l^{\tau }\right\}}_{\tau =1}^{t}\right)=Pr\left(b_{j}^{t+1}=b\;|\;b^{t}\right)=\underset{l\;|\;b=b_{j}^{t+1}\left(b^{t},l\right)}{\sum }Pr\left(l^{t+1}=l\right)$$

*where we define*

(8) $$b_{j}^{t+1}\left(b^{t},l\right)≜\frac{\sum_{i}T_{ij}b_{i}^{t}f\left(l^{t+1},j\right)}{\sum_{k}\sum_{m}f\left(l^{t+1},k\right)T_{mk}b_{m}^{t}}.$$

**Proof.**  Observe that

(9) $$\begin{array}{l} Pr\left(h^{t+1}=j\;|\;{\left\{l^{\tau }\right\}}_{\tau =1}^{t}\right)\\ \;=\underset{i}{\sum }Pr\left(h^{t+1}=j\;|\;h^{t}=i,{\left\{l^{\tau }\right\}}_{\tau =1}^{t}\right)\cdot Pr(h^{t}=i\;|\;{\left\{l^{\tau }\right\}}_{\tau =1}^{t})\underset{\left(a\right)}{=}\\ \;\underset{i}{\sum }Pr(h^{t+1}=j\;|\;h^{t}=i)\cdot Pr(h^{t}=i\;|\;{\left\{l^{\tau }\right\}}_{\tau =1}^{t})≜\underset{i}{\sum }T_{ij}b_{i}^{t}, \end{array}$$

where (*a*) uses the assumption that h is a Markov chain. In a similar manner, given the belief state $b^{t}$, the next set of sensor outputs, $l^{t+1}$, is independent of the past sensor outputs:

(10) $$\begin{array}{c} Pr\left(l^{t+1}\;|\;{\left\{l^{\tau }\right\}}_{\tau =1}^{t}\right)=\underset{j}{\sum }Pr\left(l^{t+1}\;|\;l_{1:t},h^{t+1}=j\right)\cdot Pr\left(h^{t+1}=j\;|\;{\left\{l^{\tau }\right\}}_{\tau =1}^{t}\right)=\\ \underset{j}{\sum }\mathrm{Pr}\left(l^{t+1}\;|\;h^{t+1}=j\right)\cdot \mathrm{Pr}\left(h^{t+1}=j\;|\;{\left\{l^{\tau }\right\}}_{\tau =1}^{t}\right)=\underset{j}{\sum }f\left(l^{t+1},j\right)\cdot \\ \mathrm{Pr}\left(h^{t+1}=j\;|\;{\left\{l^{\tau }\right\}}_{\tau =1}^{t}\right)=\underset{j}{\sum }\underset{i}{\sum }f\left(l^{t+1},j\right)T_{ij}b_{i}^{t} \end{array}$$

This shows that the belief state summarizes all relevant information given past sensor outputs. Furthermore, the belief state can be recursively updated by:

(11) $$\begin{array}{l} b_{j}^{t+1}=\mathrm{Pr}\left(h^{t+1}=j\;|\;{\left\{l^{\tau }\right\}}_{\tau =1}^{t+1}\right)=\frac{\mathrm{Pr}\left(h^{t+1}=j,\;l^{t+1}|\;{\left\{l^{\tau }\right\}}_{\tau =1}^{t}\right)}{\mathrm{Pr}(l^{t+1}|\;{\left\{l^{\tau }\right\}}_{\tau =1}^{t})}\\ \;=\frac{\mathrm{Pr}\left(h^{t+1}=j\;|\;{\left\{l^{\tau }\right\}}_{\tau =1}^{t}\right)\cdot \mathrm{Pr}\left(l^{t+1}\;|\;h^{t+1}=j,\;{\left\{l^{\tau }\right\}}_{\tau =1}^{t}\right)}{\mathrm{Pr}\left(l^{t+1}|\;{\left\{l^{\tau }\right\}}_{\tau =1}^{t}\right)}\underset{\left(a\right)}{=}\\ \;\frac{\sum_{i}T_{ij}b_{i}^{t}f\left(l^{t+1},j\right)}{Pr(l^{t+1}\;|\;{\left\{l^{\tau }\right\}}_{\tau =1}^{t})}\underset{\left(b\right)}{=}\frac{\sum_{i}T_{ij}b_{i}^{t}f\left(l^{t+1},j\right)}{\sum_{k}\sum_{m}f\left(l^{t+1},k\right)T_{mk}b_{m}^{t}} \end{array}$$

where (*a*) follows from Equation (9) and, since$\;b_{j}^{t}=\mathrm{Pr}\left(h^{t}=j\;|\;{\left\{l^{\tau }\right\}}_{\tau =1}^{t}\right)$ by definition, (b) follows from Equation (10). □

Lemma 1 can be straightforwardly applied to simplify the calculation of the misclassification costs:

**Corollary** **1.** *The misclassification costs can be written as*

(12) $$\rho_{j}\left(b^{t+1}\right)=\rho_{j}\left(b^{t},l\right)=C_{FP}\sum_{i=1}^{j-1}b_{i}^{t+1}\left(b^{t},l\right)+C_{FN}\sum_{i=j+1}^{J}b_{i}^{t+1}\left(b^{t},l\right)$$

*so the controller can calculate the total estimated misclassification cost as follows:*

(13) $$\rho \left(b^{t},l\right)=\sum_{j=1}^{J}b_{j}^{t+1}\left(b^{t},l\right)\cdot \rho_{j}\left(b^{t},l\right).$$

## 4. Sensor Activation Control

The Markovity of the belief states allows us to pose our problem as a MDP with the belief state as the system state. As opposed to tracking all past measurements, the belief state maintains its dimension over time. This enables us to formalize the dynamic programming equations, resulting in a tractable approximation of the optimal policy. In this section, we discuss two control policies that are based on the belief state MDP. The first is the optimal policy (Section 4.1), and the second is a one-step look-ahead greedy policy (Section 4.2). We prove that with accurate sensors that are all activated or deactivated together, the optimal policy can be computed accurately and efficiently (Section 4.3). 

### 4.1. Optimal Policy 

The Markov property of the belief states proven in Lemma 1 shows that our problem can be formulated as a MDP over a finite time horizon of L epochs. Define the value function $V^{o}\left(b^{t}\right)$, which is the minimum total expected cost of the path that starts at $b^{t}$, also known as the “cost to go” [21]. Then, the optimal solution is given by the following dynamic programming equation on $V^{o}\left(b^{t}\right)$:

(14) $$V^{o}\left(b^{t}\right)=\underset{s^{t}}{\min}\left\{\left(1-\omega \right)C\left(s^{t}\right)+\int_{b}\left(Pr\left(b^{t+1}\;=b|\;b^{t}\right)V^{o}\left(b^{t+1}\right)+\omega \rho_{j}\left(b\right)\right)db\right\}\;\underset{\left(a\right)}{=}\underset{s^{t}}{\min}\left\{\left(1-\omega \right)C\left(s^{t}\right)+\underset{l\in L\left(s^{t}\right)}{\sum }\left(Pr\left(l^{t+1}=l\;|\;b^{t}\right)\left(V^{o}\left(b^{t+1}\left(b^{t},l\right)\right)+\omega \rho_{j}\left(b^{t}\right)\right)\right)\right\}$$

where (*a*) follows from Equation (10), which also shows that

(15) $$Pr\left(b^{t+1}\left(b^{t},l\right)=b\;|\;b^{t}\right)=Pr\left(l^{t+1}=l\;|\;b^{t}\right).$$

Here, ω is the weight the system designer assigns on misclassification costs vs. power costs. Lower values of ω result in emphasizing power cost reduction, while higher values result in minimizing misclassification costs. The default value of ω in our experiments is 0.5.

From Equation (14), we can extract an optimal policy by selecting the $s^{t}$ that minimizes the value function for the belief state, i.e., $\underset{s^{t}}{\mathrm{argmin}}\;V^{o}\left(b^{t}\right)$. Equation (14) can be solved using the value iteration method [21].

Our problem can be formulated with an infinite horizon by adding a discount factor to Equation (14). Alternatively, we can assume a terminal health state, which acts as an absorbing state in the Markov chain. In practice, this state occurs when the WBAN health sensing is no longer relevant: for example, when the patient arrives at an emergency room and requires medical intervention, hospitalization, mortality, etc. 

### 4.2. One-Step Look-Ahead Greedy Policy

The greedy algorithm is a one-step look-ahead policy that at each epoch finds which sensors to activate in order to minimize the immediate cost incurred. The value function of this greedy policy is:

(16) $$V^{g}\left(b^{t}\right)=\underset{s^{t}}{\min}\left\{\left(1-\omega \right)\cdot C\left(s^{t}\right)+\omega \cdot \sum_{l\in L\left(s^{t}\right)}Pr\left(l^{t+1}=l|b^{t}\right)\cdot \rho \left(b^{t},l\right)\;\right\}.$$

### 4.3. Accurate Sensor Use Case

In this subsection, we consider the case of accurate binary sensors, which can either all be activated or deactivated at a certain epoch. Although in general, sensors are not accurate, it is reasonable to assume that sensors manufactured for tracking health states will be fairly accurate. The action space is S={0,1}, such that all sensors are activated if a=1 and idle if a=0. The sensor output probabilities are $A_{nj}\in \left\{0,1\right\}$ for all n,j. We also note the since we assume binary sensors, a single sensor output probability matrix may be used. In addition, we assume that the sensor output probabilities matrix is invertible, which can be achieved by design. As a result, each possible sensor output vector maps to a single health state. We note that when a single mapping exists between each possible sensor output vector and each health state, the problem can be solved regardless of ***A***’s structure. The following lemma shows that in this case, the number of effective belief states is finite and linear in both the time horizon L and the number of health states J. Hence, the dynamic programming problem can be accurately solved.

**Lemma** **2.** *Let* S={0,1}*, and let the sensors be fully accurate, where* $A_{nj}\in \left\{0,1\right\}$ *for all* n,j*. Assume that* A *is invertible. Then, starting from the belief state* $b^{0}$*, only* L·(J+1)*belief states are reachable.*

**Proof** **of** **Lemma** **2.** Let $h^{t}$ be the health state at time t and $h^{t}$ be the standard vector that has one at the $h^{t}$-th component. If the matrix A is invertible, then we can reconstruct the health state as $h^{t}=A^{-1}l$. Hence, $f\left(l,k\right)=1_{\left\{k=A^{-1}l\right\}}$. For $j\neq h^{t+1}$, we have $b_{j}^{t+1}=0$, and for $j=h^{t+1}$

(17) $$b_{j}^{t+1}=\frac{\sum_{i}T_{ij}b_{i}^{t}f\left(l^{t+1},j\right)}{\sum_{k}\sum_{m}f\left(l^{t+1},k\right)T_{mk}b_{m}^{t}}=\frac{\sum_{i}T_{ij}b_{i}^{t}1_{\left\{j=A^{-1}l\right\}}}{\sum_{k}\sum_{m}1_{\left\{k=A^{-1}l\right\}}T_{mk}b_{m}^{t}}=\frac{\sum_{i}T_{ij}b_{i}^{t}}{\sum_{m}T_{mk}b_{m}^{t}}=1.$$

If the sensors are not activated (i.e., a=0), f(l,k)=1 for all l; hence,

(18) $$b_{j}^{t+1}=\frac{\sum_{i}T_{ij}b_{i}^{t}}{\sum_{k}\sum_{m}T_{mk}b_{m}^{t}}\underset{\left(a\right)}{=}\underset{i}{\sum }T_{ij}b_{i}^{t}\Rightarrow b^{t+1}=T\cdot b^{t}$$

where (a) follows since the denominator sums over the entire probability space. To summarize:

(19) $$b^{t+1}=\{\begin{array}{c} T\cdot b^{t}\;\;\;\;\;a=0\\ h^{t+1}\;\;\;\;a=1 \end{array}\;$$

which means that if all sensors are activated, we can perfectly estimate the health state $h^{t}$. Hence, only belief states ${ℬ}_{0}=\{b|b=T^{m}\cdot b^{0}\;\mathrm{or}\;b=T^{m}\cdot h\;\mathrm{for}\;m\in \mathbb{N},\;h\in J\}\;$are reachable from $b^{0}$, where J is the set of J pure health states. The set ${ℬ}_{0}$ can be computed in advance before solving the dynamic programming equation. □

The implication of the above result is that the dynamic programming problem can be accurately solved with complexity $O\left(L^{2}J\right)$. To implement this computation, we also exploit the fact that each belief state $b^{t}$ can transition into two possible belief states: $h^{t+1}$ and $T\cdot b^{t}$, where T is the transition matrix. 

In the general case of inaccurate sensors, the update function of $b^{t+1}$ is not quadratic or even convex. Considering the lack of a convenient structure for the problem, one needs to compute $V\left(b^{t+1}\right)$ for every belief $b^{t+1}$ in the *J*-dimensional simplex. In practice, this means that only an approximate solution can be obtained by discretizing the belief space. Therefore, the proof of Lemma 2 implies that the general scenario with inaccurate sensors or a complicated action space S can only be solved numerically. 

### 4.4. Belief State Discretization

The dynamic programming formulation in Equation (14) explores all the possible future states over a finite period. In practice, a continuous belief state results in a very large state space, which results in exponentially growing complexity in *J*. In this Subsection, we describe the simple discretization procedure for the belief state space we will use to approximate the value function $V^{o}$. The procedure first defines a finite set of valid belief state vectors. The vectors can be related to different medical treatments or protocols, as mapped by medical professionals. Then, given an actual belief state vector, the proposed procedure finds the vector from the finite set that is closest to the actual one. Finally, the procedure calculates the value function for each valid belief state vector. The steps of the procedure are the following:

1. Set a level of discretization and define the set B, which contains all valid belief state vectors.
2. Given an actual vector, b, calculate its distance $\underset{j}{\sum }\left|{\tilde{b}}_{j}-b_{j}\right|$ to each valid vector, $\tilde{b}\in B$
3. Return the vector,$\;{\tilde{b}}^{*}$ which is the closest to b.

For example, if the level of discretization is selected to be 0.2, and b=[0.17, 0.35, 0.40, 0.08], then the above procedure returns$\;{\tilde{b}}^{*}=\left[0.20,\;0.40,\;0.40,\;0.00\right]$ as the closest valid vector. After discretizing the state space, we use (14) and calculate $V^{o}\left(\tilde{b}\right)$ for each valid state $\tilde{b}\in B$.

The fine discretization required to guarantee a good approximation of the optimal policy leads to high dimensional belief space B (as we demonstrate numerically in Section 6.2). As a result, implementing the optimal policy is complicated, since the computational load grows exponentially with the dimension of B. Even solving the dynamic programming offline does not avoid the high complexity, since it requires instead storing large policy tables that map the discretized states to actions. 

To avoid discretization, function approximation can be employed, which enables solving the POMDP over a continuous state space [22], although still with high complexity. Since our focus is to study the performance of the greedy policy, we use the simple discretization method described above to provide a “very close to optimal” benchmark. 

## 5. Empirical Analysis on Glucose Data 

### 5.1. Use-Case Description

We now demonstrate the implementation of the WBAN controlling solution in a real-world use case. We use an open-source dataset of continuous glucose measurements collected from a group of diabetes type-1 patients [8]. The measurements were recorded using a wireless glucose level sensor placed on the patients for extended periods. Using our proposed greedy control policy, we aim to reduce energy consumption while maintaining high monitoring accuracies, thus extending the sensors’ lifespans and allowing longer sensing periods. The model parameters (sensor accuracies, transition probabilities, health state trajectories) were extracted from the data, allowing us to evaluate the effectiveness of the greedy policy using real-life glucose measurements that represent a potential application. 

### 5.2. The Data

The data used for this analysis were collected as part of a clinical trial aimed at assessing the efficacy of real-time glucose monitoring [8]. The raw data contain glucose measurements for a group of 232 heterogenic patients from all ages with type 1 diabetes and glycated hemoglobin (HbA1c) 7.0–10.0%. For most patients, the measurements were collected over 6 months using three different wireless real-time continuous glucose monitoring (RT-CGM) systems. 

**Figure 3:**
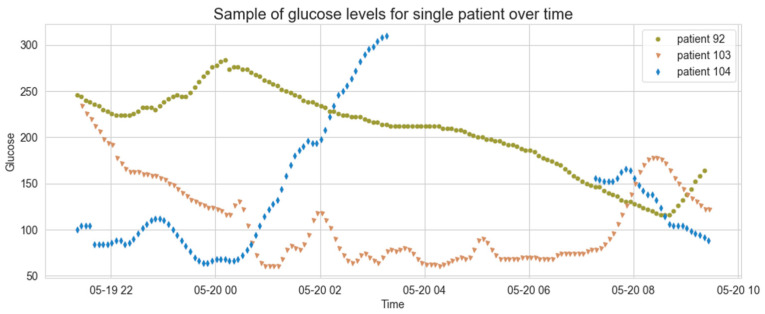
Glucose levels of randomly selected patients over time.

Figure 3 displays the glucose level measurements over time for three randomly selected patients over a period of 12 h. For the majority of patients, the time gap between subsequent measurements is 5 min. However, about 16.9% of the records have abnormal time gaps, which could be caused due to malfunctions in the measurements collection, a patient who has removed the sensor, etc. In order to overcome the difficulties when dealing with unequal gaps, we have checked what is the most common time gap per patient and set it as the patient’s time frame for measurements collection.

### 5.3. Data Modeling

#### 5.3.1. Health States

In order to apply the POMDP-based model, we first define the set of health states each patient can occupy. To this end, we discretize the range of glucose measurements by dividing the entire range of glucose values into three smaller ranges, each defined as a health state. The defined ranges are based on medical standards [23]. We note that the defined range structure can be designed according to varying medical needs. In this experiment, the belief states were chosen as $h_{3}\leq 100$, $100<h_{2}\;\leq \;180$ and $180<h_{1}$.

#### 5.3.2. Virtual Sensors and Actions

We think of the glucose data as measured using a single physical sensor, which provided a measurement every 5 min, which we call a time slot. We now describe how our model in Section 2 can be applied to control the measurement frequency of this single sensor. For this purpose, we define a time frame as k consecutive time slots. Then, one can think of each time slot as a virtual sensor and apply our model to these k virtual sensors directly. For example, if only sensor 1 is activated, then the measurement frequency would be 1/k, and if all sensors are activated, then the measurement frequency would be 1. In general, the measurement frequency does not have to be constant, since we can, for example, activate sensor 1 and sensor 2 and then not activate sensor 3 to sensor k. This way, a time frame is equivalent to the decision epoch as defined in Section 2. Hence, every time frame, the controller can change the subset set of time slots during which the single physical sensor is activated. Intuitively, the higher the sensing frequency, the more energy is consumed and the better the monitoring accuracy. The set of actions is defined by all the possible subsets of the k time slots. However, constraining the set of actions will reduce the complexity of the controller. In our experiment, we chose k=6 so that the decision time frame consists of six time slots. Then, we define the set of actions as S={(4), (2, 6), (1, 3, 5)}, where each tuple contains the index of the time slots during which the sensor will carry out a measurement. 

Then, the glucose measurements collected throughout each time frame, according to the selected action, are aggregated to a single observation using an aggregation function. The aggregated values are then discretized according to the states defined in the previous sections to provide sensor outputs l ∈ {1, 2, 3}. For example, the aggregation function may be defined as an average or maximum over the collected records. This serves as the output obtained by the k virtual sensors. In our experiment, the aggregation function is defined as the average of the collected records.

In principle, more advanced aggregation functions could be designed to average all observations throughout the decision epoch, unless there exists an observation that is relatively far from the other observations throughout the decision epoch. This would allow handling extreme observations differently than decision epochs where the observation values are relatively close to each other, promising that extreme glucose measurements are not missed due to the averaging of observations in the decision epoch. 

The system designer can select k according to varying system requirements. Smaller values of k may provide better monitoring performance, since then, controller decisions will be made more frequently. On the other hand, small values would require higher computational complexity.

#### 5.3.3. Transition Matrix

Using a portion of the patient’s measurements, the transition matrix can be estimated based on actual health state transitions. The conditional probability $T_{ij}$, is calculated as the ratio between the number of transitions between $h_{i}$ and $h_{j}$ and the number of transitions from $h_{i}$ to any of the other health states, over the selected portion. The calculated transition matrix may be used for future monitoring.

#### 5.3.4. Sensor Accuracies

The sensor output probabilities matrix is also calculated using a portion of the patient’s data. For each sensor n, we calculate the probabilities to obtain a certain output, given that the patient is in a certain health state. Since each sensor may provide an output l ∈ {1, 2, 3}, the sensor output probabilities matrix per sensor n will be of the form $A_{n}^{3\times 3}$. For example, if the patient is currently occupying $h_{1}$, the probability to obtain an output value l=2 from sensor n=3 is ${\left(A_{3}\right)}_{1,2}$.

In order to estimate the probability to receive a specific output l if the patient is in health state j, we calculate the ratio between the number of time slots where the patient was in health state j and the sensor returned an output l, divided by the number of time slots the patient was in health state j.

### 5.4. Model Parameter Extraction

Initially, we have divided the data for each patient to train and test sets to 75% and 25% of the records, respectively. Using the training set, we first extract the transition matrix and sensor accuracies per patient. Then, we use all parameters needed for the POMDP-based model (set of health states H, transition matrix T, sensor accuracies matrix A, set of possible actions S, cost parameters $C_{FN}$, $C_{FP}$) to apply a policy over the test set data. Finally, we compare the overall performance of the policy to a baseline. The baseline is defined as an inclusive policy—the sensor is activated at all time slots. This is an accuracy-optimal policy. We also compare with the dynamic programming solution, where the belief state was discretized to 9 levels to approximate the optimal policy using the value iteration algorithm [21].

Then, the collected measurements are aggregated using the aggregation function, thus creating a baseline health state prediction, for each of k time slots. To compare between policies, we will examine the total cost, the power cost, and the health state classification accuracy (calculated using the most likely health state, according to the belief state vector).

We set the following parameters: k=6, $C_{FN}=100$,$\;C_{FP}=50$ and S={(4),(2,6),(1,3,5)}, where the last one is the set of possible actions (the time slot indices in which the sensor is activated). 

The activation costs are defined to be the number of time slots where the original sensor is activated during the decision time frame of k time slots. For example, if the sensor is activated for three time slots out of a six-time slot decision time frame, the activation cost will be 3. We also note that the misclassification costs were defined to be constant for false positive and false negative scenarios, for simplicity. A more complex cost structure is applicable, such as defining increasing costs by the severity of misclassification. The use of such a cost structure will be demonstrated in Section 6.

### 5.5. Policy Comparison

Table 1 compares the overall costs of the greedy policy and the inclusive solution, averaged over all patients:

We observe that the activation costs were reduced by 51.67%, whereas the accuracy was reduced by a small factor of 1.6%, resulting in an accuracy score of 83.5%.

**Figure 4:**
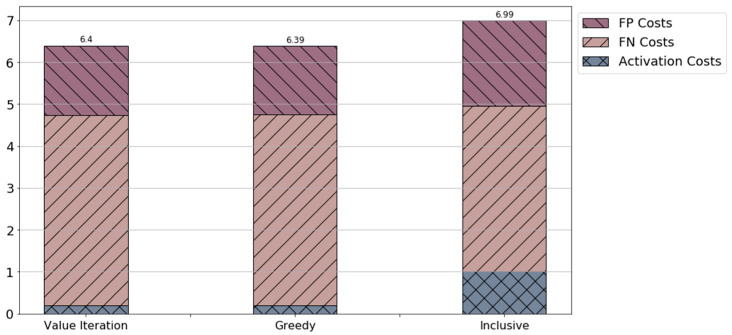
Activation and misclassification cost breakdown obtained by the different proposed policies. The costs are average over all patients. “FP” stands for false positive and “FN” stands for false negative.

Figure 4 compares the performance of value iteration (dynamic programming), greedy, and inclusive. Clearly, the inclusive policy inflicts the highest total costs. However, the misclassification costs induced by the inclusive policy are lower, compared to the greedy and value iteration solutions. In addition, the value iteration solution obtained results similar to the greedy solution. For the scenario presented here and the defined system parameters, the greedy solution provides good performance. 

Table 2 compares the false positive and false negative probabilities, averaged over all patients and time steps. The greedy solution slightly outperforms the value iteration method for both these measures. We note that this occurs mainly due to the fact that the framework aims at optimizing the total costs obtained in order to reduce activation costs, while maintaining accuracy (as presented in Table 1).

### 5.6. Sensitivity Analysis

**Figure 5:**
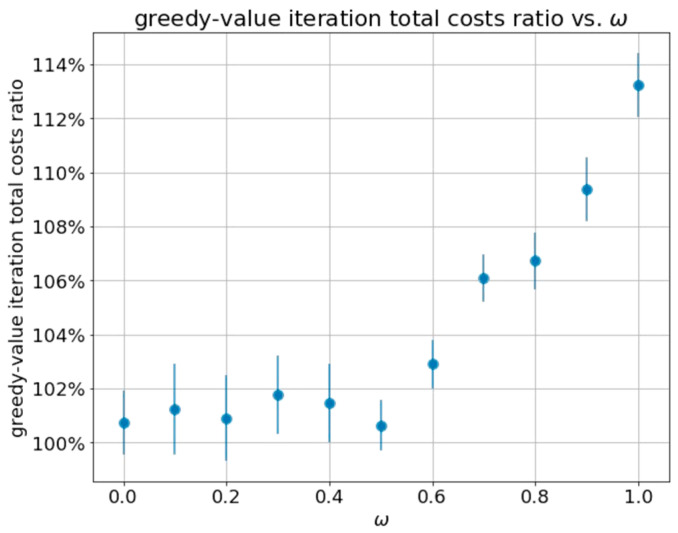
Ratio between total costs obtained by greedy and value iteration methods obtained over all patients for varying ω values, for the following set parameters: k=6, $C_{FN}=100$,$\;C_{FP}=50$.

Our results from Section 5.5 suggest that the greedy policy is nearly optimal for the scenario we extracted from the real glucose data. To establish this statement, we now conduct a sensitivity analysis of two main system parameters. We start by varying the parameter ω between 0 and 1, thus changing the weight of the two cost components in Equation (16). Lower values of ω result in emphasizing power cost reduction while higher values result in minimizing misclassification costs. Figure 5 below demonstrates the cost ratio between the greedy and value iteration methods. Each point considers the total costs obtained over all patients, and the error bars show the standard deviation of this ratio over the patients.

One can observe that the performance (total cost) of the greedy policy is never far behind the optimal policy. For the extreme case of ω=1, in which the controller will aim at minimizing misclassification costs only, the greedy method obtains costs that are approximately 13.5% higher than those of the value iteration method. However, for medical applications, the regime of interest is when the misclassification cost is more important than the energy cost since very small misclassification probabilities are required. When ω≤0.5, the cost of the greedy policy is never more than 2% more than that of the optimal policy. Hence, the greedy policy is nearly optimal for the practical regime of interest in WBAN health monitoring systems in this case. 

We next quantify the trade-off between the misclassification cost and the activation cost in the glucose use case, following the experiment above. We note that although the policy is obtained given the weighting parameter, the calculated costs reflect the true activation and misclassification costs obtained when applying the solution to the test set (i.e., without the coefficients ω and 1−ω). 

**Figure 6:**
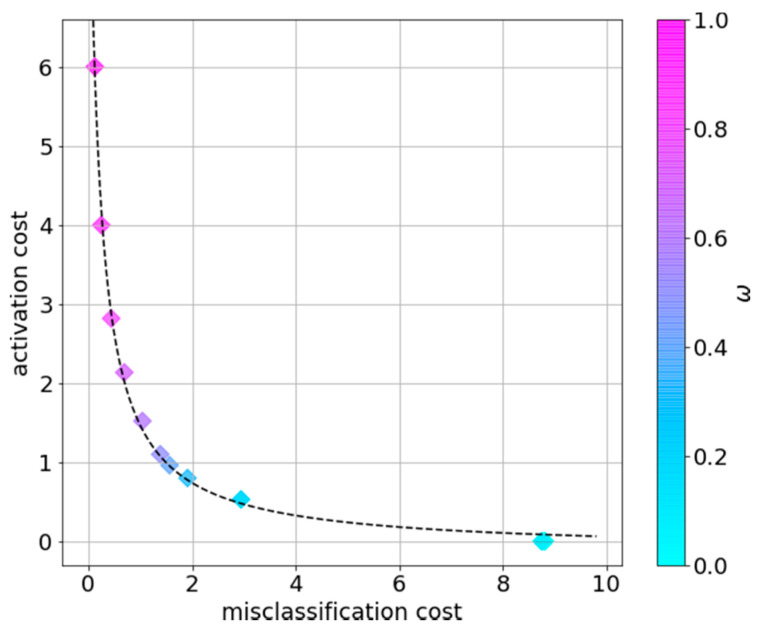
Activation costs vs. misclassification costs per ω, averaged over all patients.

The obtained data points have a high fit of $R^{2}=0.997$ to $f\left(x\right)=\frac{145.09}{276.96x+27.65}-0.07$. This high fit in Figure 6 suggests that f(x) can be used by the system designer to adjust ω for the glucose monitoring case.

**Figure 7:**
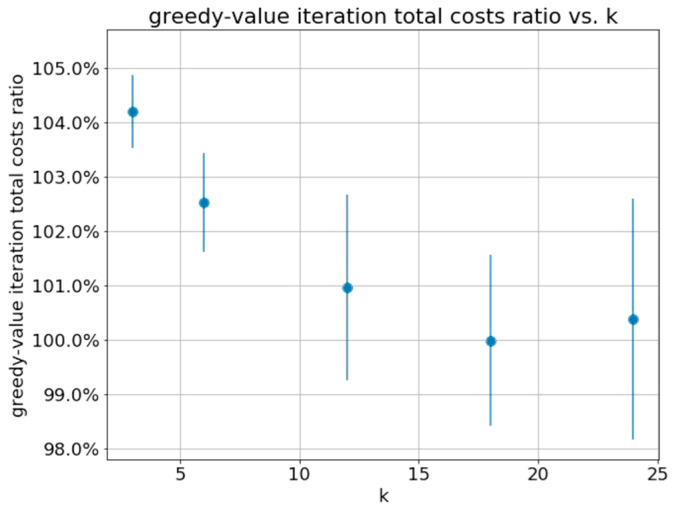
Ratio between total costs obtained by greedy and value iteration methods obtained over all patients for k values, for the following set parameters: ω=0.5, $C_{FN}=100$,$\;C_{FP}=50$.

Another main system parameter is the length of the decision epoch k. Higher values of k require lower computational complexity but will introduce more noise into the system, which is caused by the aggregation of more raw observations. Figure 7 shows that the greedy policy is nearly optimal for a wide ranges of values of k.

## 6. Empirical Analysis and Sensitivity Analysis on Synthetic Data 

In this section, we provide simple numerical examples that will allow us to study the behavior of the greedy policy compared to the optimal policy in more detail, and to provide more intuition and interpretability for the proposed greedy policy. For the glucose data, the transition matrix and sensor accuracies were estimated for the data so they do not represent a degree of freedom. Here, all parameters are arbitrary, allowing for a more general sensitivity analysis.

### 6.1. WBAN Dynamics for the Greedy Policy

We define the model parameters as follows: J=4 (the number of health states), N=5 (the number of sensors). Additional parameters are as follows:

- $T=\left[\begin{array}{cccc} 0.90 & 0.04 & 0.04 & 0.02\\ 0.02 & 0.90 & 0.04 & 0.04\\ 0.01 & 0.01 & 0.90 & 0.08\\ 0.01 & 0.01 & 0.03 & 0.95 \end{array}\right]$ is the transition matrix, from state *i* (row) to state *j* (column),
- $A=\left[\begin{array}{cccc} 0.99 & 0.01 & 0.50 & 0.50\\ 0.50 & 0.90 & 0.10 & 0.50\\ 0.50 & 0.50 & 0.90 & 0.10\\ 0.90 & 0.10 & 0.50 & 0.50\\ 0.60 & 0.40 & 0.60 & 0.50 \end{array}\right]$ is the sensor output probabilities matrix,
- $C_{s}=\left[\begin{array}{ccccc} 5 & 1 & 1 & 1 & 0.2 \end{array}\right]$ denotes the sensors’ activation costs,
- $C_{m}=\left[\begin{array}{cccc} 0 & 100 & 125 & 150\\ 25 & 0 & 75 & 100\\ 50 & 25 & 0 & 50\\ 75 & 60 & 45 & 0 \end{array}\right]$ denotes the misclassification costs where element (i, j)
- denotes the cost of misclassifying health state i as state j. Accordingly, costs above and below the diagonal represent false negative and false positive costs, respectively.

The transition matrix T was chosen based on the reasonable assumption that the probability of remaining in a certain health state is larger than the probability of transitioning between health states. In practice, the transition probabilities can be estimated using historical clinical data as we did for the glucose data in Section 5. The cost parameters were selected to reflect a clear trade-off between the misclassification costs and the sensors’ activation costs. In practice, the sensor activation costs can be based on the cost of recharging the sensors, and the misclassification costs can be estimated based on the cost of the medical care needed due to health state misclassification.

**Figure 8:**
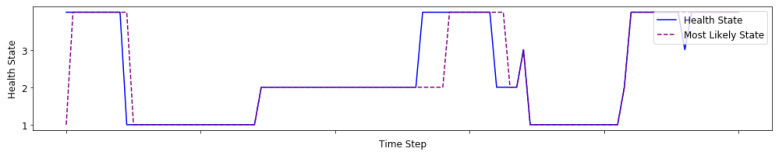
The unknown health state and the most likely health state (using the greedy policy) over 20 epochs.

We simulate the greedy algorithm described in Section 4.2 to derive a greedy policy. The most likely state shown in Figure 8 is generated by$\;h^{ML}=argmax_{j}\left(b_{j}^{t}\right)$. One can observe that the greedy policy generally provides accurate predictions of the actual health states in the proposed example.

**Figure 9:**
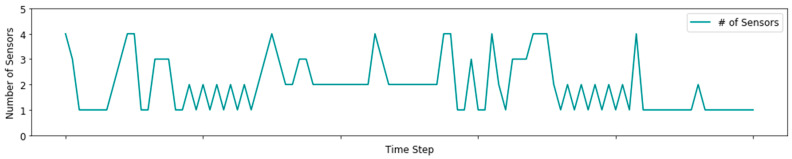
The number of sensors activated throughout the simulation using the greedy policy.

Figure 9 shows the number of sensors activated throughout the simulation. One can observe that the largest number of sensors is usually activated during health state transitions, i.e., in periods where the patient’s health is relatively unstable. In addition, Table 3 summarizes the dynamics of the activated sensors regarding their different accuracies and costs. For example, even though sensor 5 incurs low costs, it is almost never activated due to the low accuracy it provides. Sensors 1 and 4 are informative regarding $h_{1}$ and $h_{2}$. Although sensor 1 is very accurate compared to sensor 4, it is activated during a smaller number of epochs, which follows due to the lower costs incurred by sensor 4. 

### 6.2. Comparison of the Greedy Policy to the Optimal Policy 

We now compare the proposed greedy policy with the optimal dynamic programming approach, which were presented in Section 4.1 and Section 4.2, respectively. 

For this section, we define a more compact problem, which will allow deeper analysis. The parameters are defined as follows: J=3, N=2. Additional parameters are as follows:

- $T=\left[\begin{array}{ccc} 0.90 & 0.06 & 0.04\\ 0.04 & 0.90 & 0.06\\ 0.01 & 0.04 & 0.95 \end{array}\right]$ and $A=\left[\begin{array}{ccc} 0.90 & 0.10 & 0.50\\ 0.50 & 0.90 & 0.10 \end{array}\right]$
- $C_{s}=\left[\begin{array}{cc} 2 & 2 \end{array}\right]$ and $C_{m}=\left[\begin{array}{ccc} 0 & 33 & 42\\ 8 & 0 & 25\\ 17 & 8 & 0 \end{array}\right]$.

The selected policy gives the number of sensors activated given a certain belief state vector.

**Figure 10:**
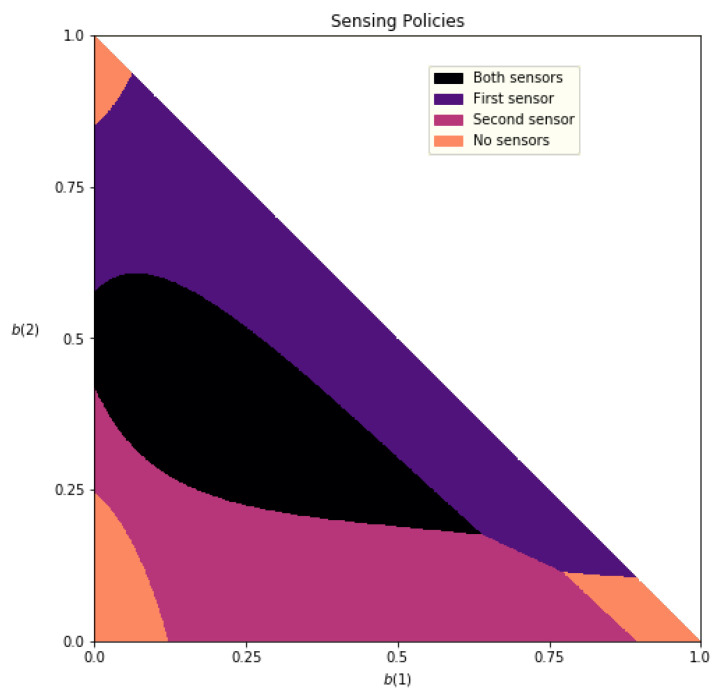
Greedy sensing policy over the belief state space b. Since $b_{3}=1-\left(b_{1}+b_{2}\right)$, the belief space forms a simplex and may be described by only the belief over the first two states.

Figure 10 maps the belief states into the subset of activated sensors. We observe that the controller tends to activate both sensors in the mid-ranges of the belief states, i.e., in areas where the information concerning the patient’s active health state is not definitive. As the belief states move toward the edges of the graph, fewer sensors are needed because there is higher certainty concerning the patient’s active health state. In the far corners of the graph, no sensors are activated. We note that the distinction between the use of the first and second sensors, when activating only one sensor, is related to each sensor’s ability to differentiate between a different pair of health states.

We now examine the different behaviors displayed by the proposed greedy policy and the dynamic programming policy. For this purpose, we use the average total costs obtained by both solutions over 30 random transition matrices as the main performance indicator. In addition, for simplicity of analysis from this point forward, we apply a simpler cost structure where the FP and FN costs are set to constant values—50 and 100, respectively. We note that all transition matrices share the property that the probabilities of remaining in certain health states are generally higher than those of transitioning to other health states. As discussed in Section 4.4, the optimal dynamic programming solution requires belief state discretization. Higher discretization resolutions will allow more accurate health state monitoring but will incur an exponentially higher computational load.

**Figure 11:**
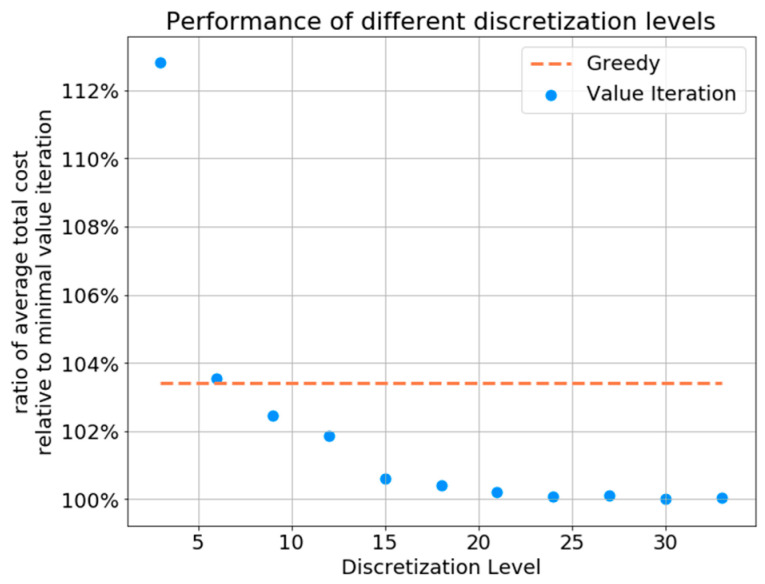
Performance of the value iteration and greedy policies as a function of the discretization level, averaged over 100 i.i.d. Monte Carlo simulations.

Figure 11 demonstrates the effect of varying the discretization resolution on the performance of the dynamic programming solution. The *x*-axis shows the “binning” resolution defined for the discretized belief state space. Higher discretization levels lead to higher accuracy when approximating actual belief states. We can see that for lower values of discretization levels, the greedy policy outperforms the dynamic programming formulation, while for higher levels, the value iteration solution outperforms the greedy solution and converges to an average total cost that is approximately 3% lower than that of the greedy solution. *Overall, the greedy solution shows a maximum gap of less than 4% from the dynamic programming solution for high discretization levels*.

We observe also that there is a downward trend up to a discretization level of 15, where the trend plateaus. From this graph, we can see that a discretization level of 15 (meaning each element in the belief state vector could be approximated by a factor of $\frac{1}{15}$) provides sufficient performance while maintaining a lower computational load compared to larger discretization levels. Hence, in the rest of the simulations, we will use 15 as the discretization level.

### 6.3. Sensor Accuracy Sensitivity Analysis

We now analyze the effect of the sensor accuracies on the policies’ performances. Generally, using sensors with poor accuracies introduces more noise into the systems that hinders their performance. We define a group of output probability matrices using a parameter ϵ as follows:

$$A=\left[\begin{array}{ccc} 1-\epsilon & \epsilon & \epsilon \\ \epsilon & 1-\epsilon & \epsilon \end{array}\right],0\leq \epsilon \leq 0.5$$

Hence, each of the two sensors differentiates between a pair of health states. Larger values of ϵ result in weaker differentiation between the pairs of health states.

**Figure 12:**
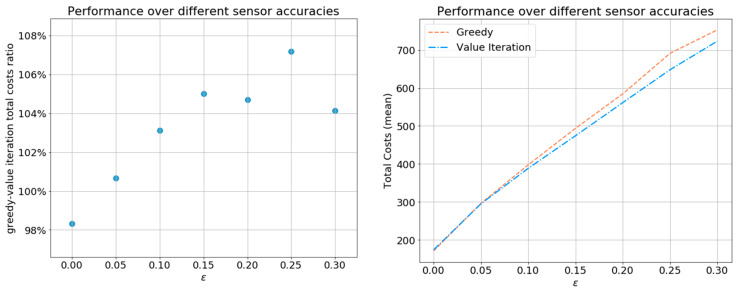
Total costs of the greedy policy and the value iteration policy over different sensor output probability matrices A defined by 0≤ϵ≤0.3 (step size 0.05), averaged over 100 i.i.d. Monte Carlo simulations.

As expected, higher values of ϵ result in higher costs for all policies, whereas near-zero values provide low costs down to the point where the sensors are perfect, which results in identical low costs for all the policies (see Figure 12). For ϵ values higher than 0.3, the sensors yield very low-accuracy outputs, causing the controller to deactivate the sensors throughout the sensing period. For the majority of examined ϵ values, the proposed greedy policy was nearly optimal. Only for very inaccurate sensors with ϵ≥0.25 is the total cost of the greedy policy more than 5% higher than that of the (approximately) optimal value iteration. This is encouraging, since medical sensors are typically required to be highly accurate in practice due to the possible implications.

### 6.4. Transition Matrix Sensitivity Analysis

Finally, we demonstrate the effect of stability in the patient’s health state trajectories on the policies’ performances. Less stable health state trajectories, in which the patient tends to change health states often, are modeled by a more equally distributed transition matrix. For that purpose, we define a group of transition matrices using a parameter $\tilde{T}$:

$$T=\left[\begin{array}{ccc} \tilde{T} & \frac{1-\tilde{T}}{2} & \frac{1-\tilde{T}}{2}\\ \frac{1-\tilde{T}}{2} & \tilde{T} & \frac{1-\tilde{T}}{2}\\ \frac{1-\tilde{T}}{2} & \frac{1-\tilde{T}}{2} & \tilde{T} \end{array}\right],0\leq \tilde{T}\leq 1.$$

**Figure 13:**
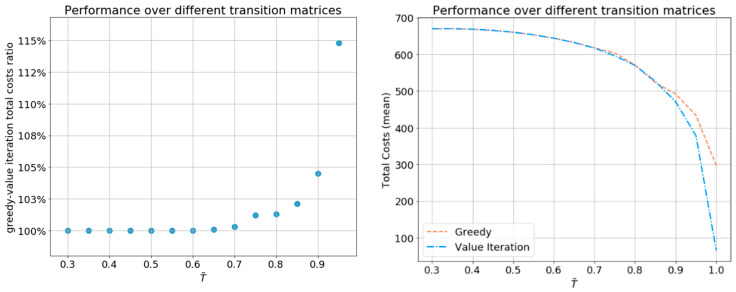
Total costs of the greedy policy and the value iteration policy over different transition matrices T, defined by $0.3\leq \tilde{T}\leq 1\;$(step size 0.05), averaged over 100 i.i.d. Monte Carlo simulations.

Note that $\tilde{T}$ determines the stability of the health state trajectories. For lower values of $\tilde{T}$, the obtained transition matrix is more uniform. For the value $\tilde{T}=0.3$, the policies yield the highest possible costs, since then, T provides no information concerning the patient’s potential health state trajectory. For higher values of $\tilde{T}$, the transition matrix T causes higher-stability health state trajectories. For the majority of $\tilde{T}$ values examined, the greedy policy is nearly optimal, showing the largest gap when the total costs of both policies decline for higher values of $\tilde{T}$. For the most extreme value of $\tilde{T}=1.0$ (not shown in the left graph in Figure 13), for which the heath state is static, the total cost of the greedy policy is 450% compared to the value iteration policy.

## 7. Conclusions

We studied the energy consumption versus accuracy trade-off in WBAN health monitoring systems. In our setting, the controller decides which subset of sensors to activate at every epoch. The objective is to monitor the unknown patient’s health state while minimizing the power and misclassification costs over time. 

We studied the performance of the simple one-step look-ahead approach for sensor activation. This simple policy is significantly easier to implement and offers better interpretability than the optimal one based on dynamic programming. Furthermore, since the optimal policy works with a continuous belief state space, it must be discretized, which degrades the performance and requires storing large policy tables. 

Our empirical study on the glucose levels of 232 patients over 6 months reveals that the proposed greedy policy saves 50% of the energy cost while losing only 1.6% of the misclassification cost compared to the policy that activates all sensors at all times (i.e., an accuracy-optimal policy). The sensitivity analysis confirms that the greedy policy is nearly optimal as long as the weight of the misclassification cost is at least twice that of the energy cost. Since in medical application, the misclassification probabilities must be very low, this weight regime includes the practical regime. 

Our findings suggest that the greedy policy can be an efficient policy to use in practice. The sensitivity analysis suggests that the reason the greedy policy is so effective is that as we estimate from the data, the health state transitions are not extremely slow, and the sensor accuracies are not extremely bad. Since this is typically the case for various medical applications, studying the performance of the greedy policy on other medical datasets will further establish the greedy policy as a practical health monitoring control for WBAN systems. 

As future research, additional cost structures can improve the applicability and real-world value of the solutions. For example, costs that dynamically change over time or different misclassification costs for more severe health states could be used. Additional extensions include generalizing for unknown POMDP parameters (transition matrix, patient health states, etc.). For example, assuming the transition matrix is unknown to the controller, sensor outputs from a group of patients may be used to initially estimate a generalized transition matrix. 

## Figures and Tables

**Table 1:** Activation costs and accuracy results.

	Activation Costs	Accuracy
Inclusive	100%	85.1%
Greedy	48.33%	83.5%
**Difference**	**−51.67%**	**−1.6%**

**Table 2:** Activation costs and accuracy results.

Misclassification Probabilities			
	Value Iteration	Greedy	Inclusive
**False Positive**	2.51%	2.47%	0.24%
**False Negative**	4.19%	4.11%	0.41%

**Table 3:** Percent of epochs during which each sensor was activated throughout the simulation.

Sensor Usage				
**1**	**2**	**3**	**4**	**5**
42%	49%	51%	55%	0%

## Data Availability

Publicly available dataset was analyzed in this study. This data can be found in the JDRF CGM study group [8]: JDRF randomized clinical trials to assess the efficacy of real-time continuous glucose monitoring in the management of type 1 diabetes: research design and methods. Diabetes technology & therapeutics, 10(4):310–321, 2008. Download: https://public.jaeb.org/datasets/diabetes (accessed on: 10 September 2019).
